# Human lungs fluid mechanics: an overview of current modelling techniques

**DOI:** 10.1140/epje/s10189-026-00583-x

**Published:** 2026-04-29

**Authors:** Francesco Romanò

**Affiliations:** Univ. Lille, CNRS, ONERA, Arts et Métiers Institute of Technology, Centrale Lille, UMR 9014-LMFL-Laboratoire de Mécanique des Fluides de Lille - Kampé de Fériet, F-59000 Lille, France

## Abstract

Fluid mechanics governs numerous physiological processes in the respiratory system, influencing airflow dynamics, particle transport and aerosol formation, airway stability, mucus transport, surfactant mechanics, and pulmonary oedema. Over the past decades, engineers, physicists, and biomedical scientists have developed a wide range of models to describe these processes across multiple spatial and temporal scales. This paper provides an integrated overview of current modelling techniques in pulmonary fluid mechanics, emphasizing the multiscale and multiphysics nature of the lung. After discussing the principal challenges in simulating the mechanics of human lungs, we review the hierarchy of modelling approaches, from first-principle continuum formulations to reduced-order and data-driven models. We then explore strategies for coupling these models and conclude with a perspective on future directions, including the need for benchmark cases and clinically robust indicators for model validation.

## Introduction

**Fig. 1:**
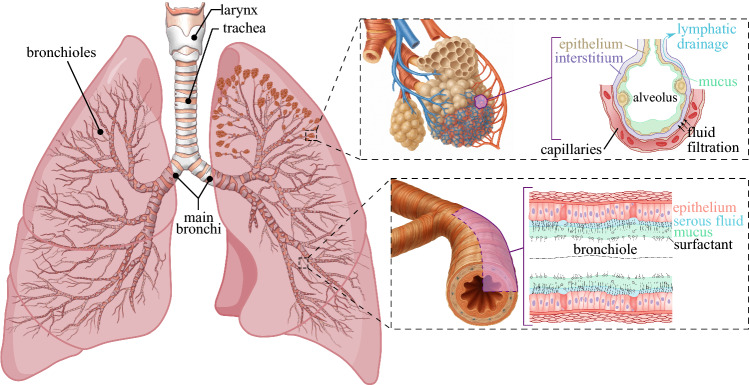
Multiscale and multiphysics schematics of adult human lung

The human respiratory system is a paradigmatic example of a living flow network in which fluid and solid mechanics interact with biochemistry over a vast range of spatial and temporal scales [1, 2] (see Fig. 1). The tracheobronchial tree forms a fractal-like branching architecture that reduces in diameter with power-law scaling $d_n = d_0\times 2^{-n/3}$ for the first 16 generations, where $d_0$ is the trachea diameter, *n* denotes the airway generation and $d_n$ is the corresponding diameter [1]. The first 16 airway generations are lined with a two-layer airway surface liquid (ASL, [3]) of thickness $h_\text {ASL,n}\approx [0.02,\ 0.04] d_n$ in healthy conditions. The corresponding flow involves three phases, i.e. air, mucus, and serous liquid (see bottom-right inset in Fig. 1), while it transitions to a two-phase system made of air and a aqueous fluid from the 17th generation on. This same generation marks the transition between the conductive zone ($n\le 16)$ and the transitional and respiratory zones ($n\ge 17)$ of adult human lungs.

The total number of bifurcations in the human lungs of an adult is 23, starting from the trachea (generation 0, internal diameter $d_0 \approx 1.5$ to 2 cm) down to the alveolar sacs (generation 23) for which $d_{23} \approx 0.08$ mm, while the alveolar diameter spans $d_a\approx 200$ $\mu$m [1, 4]. Morphometrical studies in healthy lungs report that there are $\approx 8\times 10^6$ airways [5] and $\approx 5 \times 10^8$ alveoli [6]. Thus, each respiration cycle drives fluid motion across three orders of magnitude in diameter, and the flow experiences a six-orders-of-magnitude change in cross-sectional area. This is the result of a respiratory network that evolved out of a multi-objective physiological optimization accounting for the minimization of airway resistance, uniform distribution of ventilation, and large surface for gas exchange [7]. Moreover, considering the multiphysical processes at play for the mechanics of human lungs, over six orders of magnitude are involved, spanning from the large-scale poroelastic dynamics of the respiratory cycle (an adult human lung measures $\approx 25$ cm [8, 9]) down to the small-scale blood oxygenation occurring across the lymphatic system (interstitial thickness $\approx 1$ $\mu$m [8, 10, 11]). We stress that all such morphometric references are population-averaged estimates rather than exact anatomical rules, as airway/alveoli dimensions and branching patterns exhibit substantial inter- and intra-subject variability. Nevertheless, they provide a useful reference framework for discussing the hierarchy of flow regimes and modelling approaches considered here.

Figure 1 presents a schematic illustrating the hierarchical structure of adult human lungs. Starting from the conducting airways, the generation 0 (trachea) is followed by the main or primary bronchi ($n=1$), bifurcating into lobar or secondary bronchi ($n=2$), then into segmental or tertiary bronchi ($3\le n\le 5$). The airflow in the corresponding upper generations is dominated by inertia. In the largest airways, i.e. for $d_n \gtrapprox 10$ mm for $n \le 3$, the local gas-phase Reynolds number $\text {Re}_n= U_n d_n/\nu _a$ based on the local mean airflow velocity $U_n$ and diameter $d_n$, with $\nu _a$ being the kinematic viscosity of air, frequently exceeds $10^3$ [12–15], leading to transitional or weakly turbulent jets that impinge on airway bifurcations and generate complex secondary vortices. In the remaining segmental bronchi, i.e. for $4\le n \le 5$, the flow is transitional under regular breathing conditions and weakly turbulent under deep breathing or forced respiration.

It is important to distinguish between the forces governing the airflow and those acting within the liquid lining coating the airway walls. Air motion is primarily characterized by the gas-phase Reynolds number $\text {Re}_n$, which quantifies the balance between inertial and viscous effects for the airflow. In contrast, the dynamics of the liquid layer are additionally controlled by interfacial and body-force effects that can be described through dimensionless groups such as the capillary number $\text {Ca}_n=\mu _{m} U_n/\sigma$, comparing viscous and surface tension forces, where $\mu _{m}$ denotes the mucus dynamic viscosity and $\sigma$ the mucus–air surface tension. Other relevant non-dimensional numbers are the Weber number $\text {We}_n=\rho _m U_n^2 d_n/\sigma =\text {Re}_n\times \text {Ca}_n\times (\rho _m\nu _a/\mu _m)$ (inertia versus surface tension), where $\rho _{m}$ is the mucus density, the Bond number $\text {Bo}_n=\rho _m g d_n^2/\sigma$ (gravity versus surface tension), and the Froude number $\text {Fr}_n=U_n/\sqrt{gd_n}$ (inertia versus gravity), where *g* is the gravitational acceleration. These parameters vary substantially across airway generations (see Table 1), reflecting the transition from inertia-dominated airflow in the proximal bronchi to surface tension- and viscosity-dominated regimes in the distal airways and liquid lining. For studies primarily focusing on the flow inside the liquid layer, it is generally recommended to estimate the capillary and the Weber numbers replacing the air velocity by an estimate of the liquid velocity, thereby replacing $U_n$ with $U_n \times (\mu _a/\mu _m)$.

**Table 1:** Representative dimensionless numbers across airway generations for regular breathing

*n*	$d_n$ (mm)	$\text {Re}_n$	$\text {Fr}_n$	$\text {Ca}_n$	$\text {Bo}_n$
0	15.0 – 20.0	1872 – 2496	3.59 – 7.37	0.637 – 1591	44.1 – 196
1	11.9 – 15.9	1179 – 1573	3.20 – 6.57	0.505 – 1263	27.8 – 123
2	9.45 – 12.6	743 – 991	2.85 – 5.85	0.401 – 1003	17.5 – 77.9
3	7.50 – 10.0	468 – 624	2.54 – 5.21	0.318 – 796	11.0 – 49.0
4	5.95 – 7.94	295 – 393	2.26 – 4.64	0.253 – 632	6.95 – 30.9
5	4.72 – 6.30	186 – 248	2.02 – 4.14	0.200 – 501	4.38 – 19.5
6	3.75 – 5.00	117 – 156	1.80 – 3.69	0.159 – 398	2.76 – 12.3
7	2.98 – 3.97	73.7 – 98.3	1.60 – 3.28	0.126 – 316	1.74 – 7.72
8	2.36 – 3.15	46.4 – 61.9	1.42 – 2.93	0.100 – 251	1.10 – 4.87
9	1.87 – 2.50	29.2 – 39.0	1.27 – 2.61	0.079 – 199	0.690 – 3.06
10	1.49 – 1.98	18.4 – 24.6	1.13 – 2.32	0.063 – 158	0.434 – 1.93
11	1.18 – 1.57	11.6 – 15.5	1.00 – 2.07	0.050 – 125	0.274 – 1.22
12	0.94 – 1.25	7.31 – 9.75	0.90 – 1.84	0.040 – 99.5	0.172 – 0.766
13	0.74 – 0.99	4.61 – 6.14	0.80 – 1.64	0.031 – 78.9	0.109 – 0.483
14	0.59 – 0.79	2.90 – 3.87	0.71 – 1.46	0.025 – 62.7	0.068 – 0.304
15	0.47 – 0.62	1.83 – 2.43	0.63 – 1.30	0.019 – 49.7	0.043 – 0.192
16	0.37 – 0.50	1.15 – 1.53	0.56 – 1.16	0.016 – 39.5	0.027 – 0.121
17	0.29 – 0.39	0.72 – 0.97	0.50 – 1.03	0.012 – 31.3	0.017 – 0.076
18	0.23 – 0.31	0.46 – 0.61	0.45 – 0.92	0.010 – 24.9	0.011 – 0.048
19	0.19 – 0.25	0.29 – 0.38	0.40 – 0.82	0.008 – 19.7	0.007 – 0.030
20	0.15 – 0.20	0.18 – 0.24	0.36 – 0.73	0.006 – 15.7	0.004 – 0.019
21	0.12 – 0.16	0.11 – 0.15	0.32 – 0.65	0.005 – 12.4	0.003 – 0.012
22	0.09 – 0.12	0.07 – 0.09	0.28 – 0.58	0.004 – 9.87	0.002 – 0.007
23	0.07 – 0.10	0.04 – 0.06	0.25 – 0.52	0.003 – 7.83	0.001 – 0.005

Diameters are computed following the morphometric correlation $d_n=d_0 \times 2^{-n/3}$ with $d_0\in [15,20]~\textrm{mm}$ and flow splits as $Q_n=Q_0/2^n$ with $Q_0=0.5~\mathrm {L\,s^{-1}}$, approximating the lungs as a symmetric network. Mean velocity is computed for the air flow $U_n=4Q_n/(\pi d_n^2)$. Reynolds and Froude numbers are computed for airflow as $\text {Re}_n=U_nd_n/\nu _{a}$ with $\nu _{a}(37^\circ C)=1.7\times 10^{-5}~\mathrm {m^2\,s^{-1}}$ and $\text {Fr}=U_n/\sqrt{gd_n}$ with $g=9.81~\mathrm {m\,s^{-2}}$. Capillary numbers are reported for an airway lining liquid as $\text {Ca}_n=\mu _{m} U_n/\sigma$ using $\sigma \in [0.02,0.05]~\mathrm {N\,m^{-1}}$ and $\mu _{m}\in [0.02, \ 20]~\mathrm {Pa\,s}$ accounting for healthy and pathological conditions. For studies primarily focusing on the flow inside the liquid layer, it is generally recommended to estimate the capillary number replacing the air velocity by an estimate of the liquid velocity, thereby replacing $U_n$ with $U_n \times (\mu _a/\mu _m)$. Bond numbers are $Bo_n=\rho _m g d_n^2/\sigma$ with $\rho _m=1000~\mathrm {kg\,m^{-3}}$. The selected dimensional parameter ranges reflect the variability in $d_0$, $\sigma$ and $\mu _{m}$ in adult human lungs. The non-dimensional parameters can be adjusted to different breathing flow rates $Q_0$ by multiplying $\text {Re}_n$, $\text {Fr}_n$ and $\text {Ca}_n$ by $\tilde{Q} = Q_0/(0.5~\mathrm {L\,s^{-1}})$, while $\text {Bo}_n$ remains constant across flow rates

The conducting airways continue to bifurcate leading to the subsegmental or quaternary bronchi ($6\le n\le 7$), where the diameters are $d_n\gtrapprox 2$ mm, the airflow is laminar $\text {Re}_n \approx 100$, but still affected by significant inertial effects [16]. For the quaternary bronchi and the beginning of the bronchioles, i.e. $n\ge 8$, viscous diffusion, wall compliance, surface tension, and gravitational effects in the liquid phase all contribute to the complex and potentially unstable interfacial dynamics between air and mucus [17]. Gravitational effects become negligible compared to capillary effects, i.e. $\text {Bo}= \mathcal {O}(1)$, in the bronchioles of diameter $d_n\lessapprox 2$ mm typical of $n\gtrapprox 9$ [18]. Thus, the flow in the remaining conducting airways $9 \lessapprox n \le 16$ is mainly affected by wall compliance, surface tension, surfactant, and mucus rheology [18–24], even if the respiratory cycle, i.e. $\text {We}= \mathcal {O}(1)$, could still play a significant role in stabilizing interfacial instabilities [25].

Starting from generation 12, $\text {Re}_n < 10$ in the air, and the flow in the liquid layer can closely approximate the creeping flow regime $\text {Re}_n/ \tilde{\nu }\ll 1$, where $\tilde{\nu }\in [1,\ 1000]$ denotes the liquid-to-gas kinematic viscosity ratio. For $n \ge 12$, the airway flow in the liquid layer can therefore be quasi-steady under normal breathing conditions, while it results in a low-Reynolds-number regime for the gas phase. Finally, in the respiratory bronchioles ($17\le n \le 19$), the alveolar ducts ($20\le n \le 22$) and the alveolar sacs (acinar airways, $n=23$), the airway flow is significantly more affected by wall compliance, surface tension and gas exchange with the alveolar sacs, while rheology turns out to be well approximated by the Newtonian constitutive model [26, 27]. Under quiet breathing conditions, i.e. $\tilde{Q} = Q_0/(0.5~\mathrm {L\,s^{-1}})\approx 1/4$, $\text {Re}_n$ may fall below unity for $n\gtrapprox 14$, and approach Stokes-flow limit for $n\gtrapprox 19$.

Modelling efforts in pulmonary fluid mechanics broadly address three distinct, although sometimes overlapping, objectives. The first concerns the understanding of airflow and transport in healthy lungs, aimed at establishing baseline physiological behaviour and mechanistic insight. The second focuses on pathological conditions, such as airway narrowing, mucus accumulation, or tissue stiffening, to elucidate disease progression and functional impairment. The third addresses therapeutic or environmental scenarios, including aerosol drug delivery, inhalation of pollutants, or exposure to cigarette smoke, where transport and deposition processes determine clinical or health outcomes. While these categories share common physical principles, distinguishing them helps clarify the modelling assumptions and validation strategies adopted in different studies.

Understanding and predicting the dynamics of human lungs have been long-standing objectives of respiratory biomechanics and biofluid mechanics [28–31]. The challenge of modelling pulmonary flows arises precisely from this coexistence of physical regimes and from the tight coupling between air motion, tissue deformation, interfacial mechanics and biochemical transport. Indeed, comprehensive modelling should include the parenchymal tissue, composed of elastic fibres and smooth muscle, interacting mechanically with the airway pressures. On top of the various flow regimes and complex fluid structure interactions, the multiphysics to model in the lungs include surface tension that drive interfacial instabilities and the distribution of surfactant that regulate stability at the air–liquid interface. Further complexity is added by the Marangoni stresses induced by solutocapillary effects. A final note is on the coupling with other systems. In particular, the circulatory and lymphatic networks complete the coupling between airflow, tissue deformation, and fluid transport and oxygenation. This multiscale coupling across physical domains motivates the modelling approaches and coupling frameworks discussed throughout the paper.

### Historical overview

Early physiological studies in the middle of the twentieth century already recognized the importance of airway geometry in determining resistance and gas distribution. The first morphometric analysis [1] provided quantitative description of the airway tree, while the mechanical measurements between the 1950s and early 1970s established the pressure–volume behaviour of the lung and the concept of regional compliance [32–35]. Theoretical analyses in the 1960s–1980s employed lubrication theory [36–38], linearized elasticity [39], and simple network analogues [40] to approximate airway resistance and compliance [41–43], while at the beginning of the early 1980s, these observations have been framed in the language of continuum mechanics, presenting the lung as a nonlinear, nearly incompressible material whose microstructure could be idealized as a network of elastin–collagen fibres embedded in a fluid-filled matrix [2].

By the late 1980s and early 1990s, developments in experimental fluid mechanics allowed direct visualization of airflow using particle-image [44] and laser-Doppler velocimetry [45]. These experiments revealed secondary vortices and asymmetric velocity profiles even in nominally symmetric bifurcations, recirculations, and unsteady vortex structures at bifurcations even during normal breathing [13, 46–49]. These experiments, together with theoretical work on branching networks [50, 51], established that flow in the upper lung is inherently unsteady and carries strong three-dimensional features. Such studies also provided a mechanistic basis for interpreting inhaled aerosol deposition during inspiratory [52] and expiratory deposition [53].

The progression from these physical models to modern image-based computation was made possible by advances in medical imaging. High-resolution computed tomography (CT [54]) and magnetic-resonance imaging (MRI [55]) now permit reconstruction of the airway tree down to diameters of about one millimetre, corresponding to the eighth or ninth generation [28, 56, 57]. This technological shift enabled subject-specific computational fluid dynamics (CFD) simulations of breathing flows, capable of capturing realistic boundary conditions and wall motion [16, 56, 58]. CFD studies have since characterized inspiratory and expiratory flow patterns [59], wall shear stresses [60], turbulence transition [61], and the distribution of aerosols used in inhalation therapy [15, 29]. They also clarified how geometry and curvature promote local turbulence and how such effects influence aerosol deposition [62].

Complementary to large-airway investigations, other simulations focused on the deep lungs. Such simulations demonstrate that the physics of breathing in distal airways cannot be understood by considering air as the sole phase to simulate: the influence of tissue motion [63–65], surfactant and air-ASL interfacial dynamics [18, 21, 23, 66], mucus rheology [22, 67, 68], ciliary motion [24, 69, 70], and the generalized elastic properties of the airway wall [71, 72] must be incorporated even for a qualitative reproduction of observed phenomena.

The last two decades have seen an increasing growth in the use of numerical simulation for pulmonary research. As computing power increased and algorithms matured, models of airway flow progressed from rigid-wall, steady-state calculations to fully coupled, time-dependent multiphase simulations of the breathing cycle. Large-eddy simulations (LES), hybrid Reynolds-averaged/LES approaches, and direct numerical simulations have been used to explore turbulent structures in the trachea, the main bronchi [15, 29, 73], and distal airways [74, 75], while lattice-Boltzmann and immersed-boundary methods provided efficient alternatives for complex geometries and moving boundaries [16, 58]. Validation against magnetic-resonance velocimetry and PIV measurements shows that modern simulations in the upper airways can reproduce measured mean velocities and pressure distributions within about 10–20% when geometry and boundary conditions are well matched [14, 76]. The importance of accurate geometric modelling for the upper lungs is further confirmed by comparisons with in vivo deposition, pointing out that accurate representation of the mouth–throat region and inhalation waveform are crucial [62, 76]. Complementary to these computational efforts, several in vitro experiments have been developed to investigate the mechanics of the distal lung region. They studied the mucociliary clearance [77, 78] and investigated the formation of clean and surfactant-laden liquid plugs (airway closure [79, 80]), their propagation and rupture [81], as well as the corresponding response of epithelial cells [82].

While most models assumed rigid walls, a few studies incorporated wall compliance and interaction with surrounding parenchyma through fluid–structure-interaction (FSI) frameworks [83, 84]. These models reproduce phenomena observed clinically, such as wheezing and acoustic interactions and cyclic airway narrowing and reopening, and reveal how transmural pressure and tissue stiffness modulate regional ventilation [58, 85–87]. Poroelastic formulations extend this idea by treating the parenchyma as a deformable porous continuum that exchanges air with compliant airways, reproducing hysteresis and heterogeneity seen in imaging [87, 88]. When coupled with poroelastic descriptions of tissue deformation, such computational models form the foundation of current efforts to simulate an entire breathing cycle with realistic physiology [89, 90].

A parallel effort focused on the integration of multiscale coupling into these computational frameworks. Because direct simulation of all 23 generations of airways is infeasible, hybrid 3D–1D–0D approaches link high-fidelity CFD in the proximal airways with reduced-order network representations of the distal tree [91–94]. Each distal outlet of the 3D domain is attached to a one-dimensional branch governed by unsteady Poiseuille or Womersley flow, and the terminal units are represented as lumped compliances. This hierarchy ensures physiological boundary conditions and permits comparison with imaging or spirometric data [95, 96]. Recent implementations have extended the concept to include perfusion and gas-exchange models, thereby coupling ventilation with blood flow and oxygen transport [97, 98].

The convergence of imaging and computation has led to the concept of the “digital lung twin”: a patient-specific virtual model that can predict the mechanical and physiological response of an individual’s respiratory system to interventions such as aerosol therapy or mechanical ventilation [28, 58, 99]. These digital twins rely on accurate representation of the underlying physics, robust parameter estimation from data, and efficient numerical solvers. They also highlight the importance of uncertainty quantification and sensitivity analysis, since even small errors in geometric or material parameters can alter global predictions [100]. Machine-learning surrogates, trained on high-fidelity CFD or FSI data, can now approximate pressure–flow relationships and particle deposition within milliseconds, making near-real-time clinical prediction conceivable [100]. Such integration of data and physics signals a shift from purely mechanistic modelling to hybrid, data-assimilated frameworks.

Despite recent advances, a digital twin of the deep lungs remains far from predictive. In the distal region, airflow strongly couples with tissue deformation, ASL-film and surfactant dynamics, and local biochemical processes, making multiphysics modelling inherently complex. Current frameworks cannot yet capture this interplay at realistic scales or with sufficient experimental validation. While digital twins of the conducting airways are becoming clinically relevant, extending such fidelity to the acinar level will demand significant advances in multiscale coupling and data-driven model calibration.

The behaviour of the airway surface liquid, for instance, has been described using viscoelastic or viscoplastic constitutive laws derived from rheological measurements [23, 24, 101, 102]. Some of these studies connect directly with mucociliary clearance, the first line of defence of the respiratory tract, in which coordinated ciliary beating propels mucus and trapped particles towards the pharynx [103]. Thin-film models incorporating yield stress and surface tension have reproduced the formation and rupture of liquid plugs, processes associated with airway reopening and closure in disease and during mechanical ventilation [19, 30]. At the same time, alveolar models coupling surface tension mechanics with surfactant kinetics have explained how the lungs maintain stability despite the enormous number of individual air spaces [104]. The integration of such microscale physics into organ-scale simulations remains an active frontier.

Clinically, the relevance of these models has expanded from research to therapy. Bronchoconstriction and airway wall thickening modify the local Reynolds number and promote recirculation; changes in mucus viscosity and surface tension alter clearance and stability; and mechanical ventilation imposes pressure oscillations that can injure tissue through cyclic over-distension. Specifically, in chronic obstructive pulmonary disease (COPD) and asthma, simulations clarify how airway narrowing and wall thickening change local Reynolds numbers and promote recirculation and gas trapping [105]. Accurate prediction of flow redistribution and gas trapping can inform both diagnosis and treatment planning. In cystic fibrosis, abnormal mucus rheology leads to impaired clearance and infection; non-Newtonian mucus rheology has been modelled to quantify how changes in viscosity, yield stress, and viscoelasticity affect mucociliary transport altering clearance rates [24, 106].

For acute respiratory distress syndrome (ARDS) and neonatal surfactant deficiency, models of alveolar recruitment, surfactant transport, and thin-film dynamics explain alveolar collapse and reopening during mechanical ventilation, helping to optimize surfactant replacement therapy [19, 30, 104, 105]. Aerosol transport and deposition constitute a general physical problem that underlies several applications. In therapeutic contexts, these models support the optimization of inhaled drug delivery by helping clinicians tailor particle size and inhalation profiles to maximize deposition in targeted regions [16, 62, 73]. In other contexts, similar modelling approaches are used to assess exposure to airborne pollutants or cigarette smoke [107, 108]. Although governed by the same transport mechanisms, the objectives and modelling assumptions differ across these applications. Thus, the synergy between fluid mechanics and clinical respiratory medicine has become increasingly reciprocal: clinical observations pose quantitative challenges for modelling, and model predictions offer mechanistic insights that inform clinical practice.

Beyond specific diseases, the broader objective is to achieve predictive simulation of lung mechanics that can be used in a clinical workflow. Such a goal demands not only accuracy but also speed and reproducibility. To this end, machine-learning and reduced-order methods have been introduced to emulate high-fidelity solvers at a fraction of the computational cost. Neural-network surrogates trained on databases of CFD or FSI results can predict pressure–flow relationships and regional ventilation patterns within durations compatible with live model predictions [109]. Physics-informed neural networks (PINNs) and multi-fidelity Gaussian-process models are being explored for parameter estimation and data assimilation [87, 100]. When combined with uncertainty quantification frameworks, these approaches could provide the robustness required for regulatory or clinical acceptance [110].

### Scope of this overview

The progression of lung modelling, from early idealized descriptions to today’s image-based, multiphysics frameworks, mirrors a broader shift in biofluid mechanics towards integrative physiological modelling. Rather than treating the lung as a passive airflow network, current research moves towards a coupled solid and fluid mechanical system further coupled to biochemistry. The systemic function of the lungs emerges therefore from interactions between air transport, tissue deformation, surfactant behaviour, and cellular activity. The scientific challenges lie in representing these interactions consistently across scales: from laryngeal and bronchial jets to acinar recirculation, from parenchymal mechanics to regional perfusion, and from thin-film interfacial dynamics to alveolar gas exchange. Addressing the modelling approaches and how they have and could be coupled forms the rationale for the present overview.

The remainder of this article is organized as follows: Section 2 identifies and discusses the key challenges that currently limit predictive modelling of the respiratory system, including geometric complexity, multiphase effects, nonlinear material behaviour, data scarcity, and computational cost. Section 3 surveys the principal classes of models employed in the literature, from first-principle continuum formulations and fluid–structure interaction to reduced-order network representations, poroelastic frameworks, interfacial thin-film models, and data-driven surrogates. Section 4 then reviews strategies for coupling these models across scales and physics, emphasizing hierarchical 3D–1D–0D frameworks, fluid–structure and airflow–perfusion coupling, and algorithmic considerations for stability and efficiency. Finally, Sect. 5 offers perspectives on future directions, benchmark development, and clinically robust indicators that could bridge the gap between model prediction and patient care.

## Modelling challenges

This section summarizes the main physical constraints and modelling challenges that arise across airway generations, providing the basis for the modelling strategies discussed in Sect. 3. Despite the significant progress in both experimental and computational respiratory research over the last decades, the simulation of lung mechanics and transport processes remains a scientific and technical challenge. The lung is a dynamic, heterogeneous, multiphase system driven by cyclic boundary conditions and embedded within a complex chest-wall geometry [28, 58]. This section summarizes the main physical, numerical, and physiological challenges that currently limit predictive modelling.

### Geometric complexity and variability

The geometry of the tracheobronchial tree is neither symmetric nor self-similar beyond a few generations. Imaging studies reveal large inter- and intra-subject variability in branching angles, diameters, and lengths [1, 43, 54, 58]. Even in healthy individuals, airway calibre depends on posture, lung volume, and smooth-muscle tone [111–113]; disease adds further irregularities through wall thickening, mucus plugs, and parenchymal destruction [114–116]. Reconstructing and meshing this highly irregular structure for numerical simulations is computationally intensive and prone to discretization artefacts [117]. For this reason, well-defined benchtop experiments provide a necessary stepping stone for validation of modelling approaches [76]. Segmentation of distal airways from CT or MRI typically resolves only the first eight to ten generations; the remaining acinar structure must be inferred statistically [28].

Errors in geometry propagate into pressure–flow relationships, resistance, and predicted deposition patterns, emphasizing the need for stochastic or uncertainty-aware representations of airway morphology [28, 100]. Pathological changes, such as airway wall thickening, mucus plug formation, or alveolar destruction, introduce additional geometric irregularities that further limit the predictability of numerical and theoretical models.

### Regime transitions and multiphase effects

The coexistence of laminar, transitional, and creeping flows within a single breathing cycle creates major modelling difficulties. Turbulence models tuned for engineering flows may fail to capture the transitional vortices and secondary flows typical of the trachea and main bronchi [15, 29, 46]. For distal airways and alveoli, inertia progressively looses significance and unsteady laminar flows dominates before the creeping flow regime is met.

Moreover, thin films of mucus and surfactant introduce additional phases with complex rheology and surface tension gradients [19, 23, 102]. Accurate representation of these films requires coupling free-surface dynamics, surface tension, and interfacial transport, often through moving boundaries or interface-tracking/-capturing methods. Film rupture, coalescence, and yield-stress behaviour produce discontinuities that challenge standard numerical schemes [22, 23, 74, 75, 118]. Moreover, airways may collapse when surface tension and elastic forces overcome transmural pressure, introducing topological changes in domain connectivity that are difficult to track numerically [30, 64, 86], especially when linear instabilities are identified. The exponential growth predicted by linear stability analysis for small perturbations highlights the sensitivity of these systems to neglected nonlinear effects and further stresses the need for fully nonlinear modelling approaches.

### Nonlinear tissue mechanics and coupling stiffness

The parenchyma, i.e. the soft and compliant tissue composed of alveoli, alveolar ducts, and the surrounding interstitium, exhibits highly nonlinear, anisotropic mechanical behaviour arising from the recruitment and stretching of collagen and elastin fibres. At small strains, the tissue is compliant; at larger deformations, stiffness increases sharply [2, 87]. This nonlinear elasticity couples bidirectionally with the airway pressures that drive ventilation, producing stiff systems of equations when solved simultaneously with fluid flow [83, 88].

Partitioned FSI algorithms can become unstable if coupling is weak or time steps are too large; strongly-coupled schemes, while robust, demand high memory and computational cost. Furthermore, constitutive parameters such as fibre orientation, bulk modulus, and damping are unavailable for individual subjects. Experimental measurements are often performed on excised tissue or animal models under conditions that differ from *in vivo* loading, leading to uncertainty in parameter calibration [87, 119].

### Temporal and spatial scale separation

Processes in the lung span milliseconds to minutes and micrometres to tens of centimetres [1, 120, 121]. The ratio between Reynolds numbers suggests that turbulent eddies in the trachea evolve tens of thousands times faster than alveolar deformation. Because airway dynamics involve many interacting processes spanning this entire range of frequencies, with no clear separation between dominant time scales, fully resolving all these dynamics simultaneously is currently impossible. Adaptive time-stepping and multi-rate integration can alleviate stiffness, but coupling between fast and slow processes may still produce numerical instability and does not seem achievable in the near future.

Spatially, the contrast between the centimetre-scale trachea and micrometre-scale alveolar septa implies a ratio of $10^4$ to $10^5$ in characteristic length. Further considering that the multilayer liquid-film coating (encompassing mucus and serous layers) is two orders of magnitude smaller than the corresponding airway radius, the spatially multiscale problem almost ubiquitously spans over four orders of magnitude in the vast majority of the human lungs. Uniform resolution of such scales would require trillions of grid points, rendering direct numerical approaches technically unapproachable.

Hybrid 3D–1D–0D and homogenization approaches therefore remain essential but introduce new questions about how to close sub-grid models and how to propagate uncertainties from one scale to another [28, 93, 122]. Such multiscale coupling introduces closure problems and numerical stiffness that remain active research topics [31, 58].

### Data scarcity and model validation

Comprehensive validation of lung models is hindered by the scarcity of quantitative experimental data. Direct measurements of flow velocity and pressure inside living lungs (so-called *in vivo* measurements) are limited by invasiveness and spatial resolution. Non-invasive imaging such as phase-contrast MRI provides regional ventilation but not detailed flow fields; optical methods are restricted to transparent or excised samples [123].

Consequently, most validation relies on integral quantities such as global resistance or ventilation distribution, which are insufficient to test multiphysics couplings at smaller scales. In addition, inter-subject variability and measurement noise complicate statistical comparisons between simulation and experiment.

Reproducibility suffers when codes are proprietary or when data and meshes are not publicly available. Reproducible science in this domain will require open frameworks, standardized input data, and transparent reporting of numerical parameters. Reproducibility depends on open meshes, standardized parameters, and transparent solver settings, which are still inconsistently reported [58, 76]. Open databases and benchmark geometries, similar to those established in cardiovascular modelling, are only now emerging in respiratory research. In particular, community efforts such as the SimInhale and Virtual Physiological Human consortium initiatives are now providing open geometries and benchmark data for reproducible verification [16, 28].

### Computational cost

State-of-the-art multiphysics simulations can involve tens to hundreds of millions of elements/cells/grid points, as well as implicit time stepping, and iterative coupling between fluid, structure, and surfactant transport solvers. A single breathing cycle may require hundreds of CPU hours or specialized GPU implementations. Load balancing and parallel scalability remain limiting factors, particularly when coupling distinct codes (for example, CFD and finite-element solvers for solid mechanics) developed by different communities.

Beyond raw element count, the computational burden increases substantially with the need to resolve the wide range of spatiotemporal scales present in the respiratory system. Airway flows can involve sharp jets, transitional turbulence, and thin liquid films, each requiring different mesh densities and numerical schemes. In the acinar region, resolving compliant alveolar structures and surfactant-laden interfaces introduces stiff equations and tight numerical stability constraints that require small time steps. Adaptive mesh refinement offers partial relief but also introduces overhead in mesh redistribution and memory access, limiting practical gains on modern architectures.

Coupled simulations of whole-lung function remain especially challenging because they must integrate heterogeneous models—three-dimensional CFD for proximal airways, one-dimensional networks for the conducting tree, poroelastic representations of parenchyma, and lumped alveolar units. Each subsystem evolves on distinct characteristic timescales and uses different discretization strategies, making strong coupling prohibitive. Partitioned approaches reduce implementation effort but require sub-iterations to maintain stability, adding large communication and synchronization costs on distributed computing platforms. As a result, scaling such simulations beyond a few hundred cores remains nontrivial, and full-breathing-cycle simulations remain expensive despite algorithmic and hardware advances.

### Interdisciplinary integration

Finally, successful lung modelling demands close collaboration between fluid and solid mechanicians, physiologists, and clinicians. Clinical data often come in forms unfamiliar to engineers, such as spirometric curves, CT attenuation maps, or histological measurements, while mechanical quantities such as wall stress or shear rate are rarely measured in medicine. Bridging this gap requires the translation of modelling outputs into physiologically or clinically interpretable indicators, such as regional compliance, ventilation–perfusion ratios, or risk metrics for clinically relevant events. Conversely, clinical imaging and patient monitoring can supply boundary conditions and validation data for models. Building this two-way interaction remains one of the most significant organizational challenges in the field.

Beyond these physical considerations, translating respiratory models towards predictive and clinical applications also requires robust data integration, standardized workflows, and interoperable computational infrastructures. These aspects are discussed further in Sect. 5. Taken together, the constraints outlined above define the modelling requirements across airway generations, motivating the computational approaches reviewed in the next section.

## Modelling approaches

Modelling the human lung as a coupled multiphysics, multiscale system confronts difficulties ranging from geometry and constitutive uncertainties to numerical stiffness and data scarcity. Addressing these issues requires a combination of multi-fidelity modelling techniques, efficient algorithms, and rigorous validation protocols. The following sections review how existing modelling approaches attempt to tackle these problems.

The diversity of physical regimes and characteristic scales in the lung has led to an equally diverse set of mathematical and computational strategies. Some models aim to solve the governing conservation laws of mass and momentum directly at the spatial resolution of interest; others reduce the dimensionality or the number of degrees of freedom to obtain fast, interpretable predictions suitable for clinical use. For clarity, this section reviews four major categories that have emerged in the literature: (i) first-principle models, (ii) reduced-order models, (iii) poroelastic and continuum tissue mechanics models, and (iv) data-driven and machine-learning approaches.

### First-principle models

First-principle simulations of airflow in realistic upper airway geometries represent one of the most direct application of classical fluid mechanics to respiratory physiology. They aim to solve the Navier–Stokes equations for air, possibly coupled with wall motion and minor multiphase effects, limiting simplifying assumptions and focusing on the relevance of complex geometries. The earliest numerical attempts appeared in the 1980s, when the geometry of the trachea and main bronchi was reconstructed from casts or idealized bifurcations [13]. With the advent of high-performance computing and medical imaging, three-dimensional simulations using patient-specific CT or MRI data became feasible [16, 58]. These studies established that airflow in the upper conducting airways is unsteady, three-dimensional, and highly sensitive to anatomical detail.

Modern CFD models applied to upper lungs typically employ finite-volume or finite-element discretizations on unstructured meshes containing $10^{6}$–$10^{8}$ cells. They resolve the cyclic motion of breathing by imposing time-varying inlet or outlet conditions derived from spirometry or pleural pressure measurements. In the trachea and main bronchi, Reynolds numbers range from $10^{3}$ to $10^{4}$ during quiet breathing, increasing further during forced inhalation or coughing; hence, turbulence modelling becomes essential. Large-eddy simulation and hybrid Reynolds-averaged/LES approaches have been adopted to capture transitional structures such as the laryngeal jet and secondary vortices at bifurcations [15, 29]. Validation against magnetic-resonance velocimetry and PIV measurements shows that these models in the upper airways can reproduce measured mean velocities and pressure distributions within about 10–20% when geometry and boundary conditions are well matched [76, 124]. This level of agreement typically refers to bulk quantities such as pressure drop, cross-sectional velocity profiles, and regional flow partitioning under controlled breathing conditions representative of quiet inhalation. It should also be interpreted in light of experimental uncertainties arising from imaging resolution, boundary-condition estimation, and inter-subject variability, which are often of comparable magnitude. For particle deposition studies, uncertainties may be larger due to sensitivity to particle size distribution and inhalation waveform.

Most of the present simulations are carried out without including the wall deformability. A central limitation of rigid-wall CFD is its inability to represent the compliance of airway walls and the coupling with the parenchyma. Rigid-wall assumptions are not a major concern, hence often acceptable for simulations of airflow in the upper airways, since the trachea and the main bronchi are weakly compliant [125, 126]. This approximation is primarily valid under quiet breathing conditions; during coughing, forced expiration, or other high-pressure manoeuvres, even cartilaginous airways can undergo significant deformation that alters flow patterns and pressure losses, requiring fluid–structure-interaction modelling for accurate prediction. Moving towards higher generations, the assumption of rigid airways represents an increasingly worse approximation [127, 128]. Fluid–structure-interaction formulations overcome this by solving for both the airflow and the mechanical deformation of the airway wall or surrounding tissue [64, 65, 129]. Monolithic FSI solvers treat the fluid and solid equations within a single matrix system, ensuring strong coupling but at high computational cost [130, 131]. Partitioned schemes exchange interface conditions (continuity of velocity and shear) between distinct fluid and solid solvers iteratively [132–134]. Such methods have revealed how cyclic pressure variations lead to radial wall motion, alter local resistance, and contribute to flow redistribution [86]. They also permit the study of pathophysiological mechanisms such as dynamic airway collapse, which is central to obstructive sleep apnoea and COPD.

More sophisticated models extend FSI to include poroelastic or viscoelastic tissue behaviour. The parenchyma is treated as a compressible solid saturated with air, allowing direct simulation of lung inflation and deflation [58, 87]. Parenchymal tethering plays a central role in this coupling, as the mechanical interaction between the airway wall and the surrounding alveolated tissue contributes significantly to airway stability and pressure–diameter behaviour. Experimental and theoretical studies have shown that the effective stiffness experienced by the airway depends on parenchymal shear modulus, lung volume, and surface tension effects [135–137]. Incorporating this mechanical coupling is therefore essential for realistic prediction of airway deformation and the resulting flow dynamics. Earlier morphometric and mechanical models have also provided important insight into acinar structure and heterogeneous parenchymal deformation [138, 139]. These formulations capture hysteresis in pressure–volume curves and predict spatially heterogeneous strain fields consistent with imaging observations. Recent work has further linked local tissue deformation to injury mechanisms, for example by predicting atelectasis-induced microvolutrauma during mechanical ventilation [140]. When combined with realistic boundary conditions at the pleural surface and with gravitational gradients in tissue properties, they reproduce experimentally observed ventilation distributions. Nevertheless, the computational burden remains severe: a full breathing cycle of a single-phase patient-specific FSI model may require several processor-hours even on modern clusters [100, 141], even when the multiphysics of the airway and the alveoli is oversimplified.

Another active direction is the first-principle upper-lungs simulation of multiphase flow and aerosol transport. Euler–Lagrange and Euler–Euler formulations have been used to model the trajectories, deposition, and possible re-entrainment of particles and droplets ranging from micrometre-sized therapeutic aerosols to sub-micrometre pollutants [16, 73]. These calculations have accounted for gravitational settling, inertial impaction, Brownian diffusion, and electrostatic forces, all superimposed on the unsteady breathing flow field. Comparisons with in vivo deposition data demonstrate that CFD can reproduce overall trends in deposition efficiency and regional targeting when the mouth–throat geometry and inhalation waveform are accurately represented. Coupling particle and droplet transport with humidification and heat transfer models has further enabled predictions of hygroscopic growth and the fate of airborne pathogens.

Beyond airflow, CFD and FSI frameworks have been extended to simulate blood flow in the pulmonary arteries and veins [142]. Although haemodynamics is outside the primary scope of airway mechanics, the same computational principles apply, and the coupling between perfusion and ventilation represents an important frontier. Hybrid models now integrate both domains within a single numerical framework, linking airflow in the bronchi to blood flow in the accompanying vessels. Such integrated simulations provide a foundation for studying ventilation–perfusion mismatch, a hallmark of many pulmonary diseases.

**Fig. 2:**
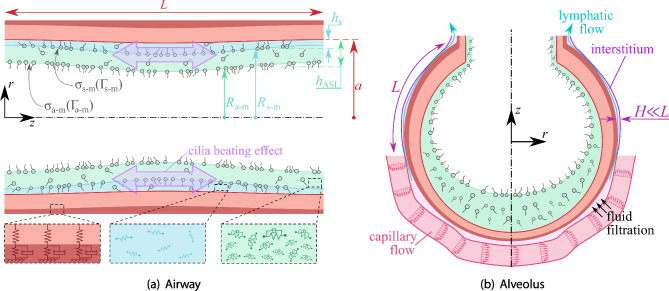
Schematic representation of **a** bronchiole and **b** alveolar multiphysics models. **a** Multiphysics in a small airway illustrating the coupling between airflow dynamics, structural deformation and poroelastic response of lung tissue, two-layer liquid coating by periciliary and mucus lining, interfacial and surfactant phenomena, ciliary beating, and non-Newtonian rheology. **b** Multiphysics in an alveolus connected to a terminal bronchiole, surrounded by capillary and lymphatic vessels embedded in the interstitium. This includes the fluid transport occurring through capillary and lymphatic flows, surface tension, and surfactant dynamics at the air–liquid interface, interstitial pressure, and tissue elasticity regulating fluid filtration and recoil

The first-principle models for deep lungs in the bronchioles are rather controlled by interface dynamics between mucus and air. In particular, thin liquid films lining the small bronchioles can undergo capillary instabilities, forming occlusive plugs (airway closure) whose subsequent rupture (airway reopening) generates large interfacial stresses capable of injuring the epithelium [82]. Airway closure and reopening constitute a highly nonlinear multiphysics problem driven by the interaction of surface tension, surfactant transport, wall deformability, and the rheology of airway surface liquid (see Fig. 2a). First-principle axisymmetric simulations have shown that surfactant gradients, viscoelastic or yield-stress properties of mucus, and compliant airway walls jointly determine plug stability, propagation speed, and reopening pressures, yielding behaviour far from that predicted by classical Newtonian models [19, 22, 74, 143, 144]. Complementary, three-dimensional models have demonstrated, through fully coupled fluid–structure formulations, that wall elasticity and airway curvature critically modulate instability onset and lead to spatially heterogeneous reopening patterns [63, 64], especially under cyclic breathing conditions. Together, these studies reveal that small-airway understanding emerges from the coupled dynamics of surfactant-laden interfaces, non-Newtonian film rheology, and deformable epithelial structures, a multiphysics that is essential to reproduce due to the extreme sensitivity of distal airflow to disease-induced changes in mucus composition, airway tissue properties and surfactant production.

For distal airways, another range of scales is also of key importance for the correct functioning of the lungs. Indeed, mucociliary clearance relies on the coordinated beating of cilia embedded in the periciliary layer (tens of microns at the airway walls), generating metachronal waves that transport mucus towards the glottis. Continuum models [69, 145, 146] have elucidated how collective ciliary forcing can be represented at tissue scale through averaged slip-velocity or active-stress boundary conditions that reproduce the emergent metachronal pattern and its wavelength dependence on hydrodynamic coupling [147, 148]. These frameworks provide mechanistic insight into how synchronization, beat asymmetry, and fluid loading shape mucus transport efficiency under physiological and pathological conditions. At the same time, coarse-grained simulations inspired by molecular-to-mesoscale modelling [149] have begun to bridge the gap between microscopic ciliary architecture, local fluid–structure interactions, and continuum-scale transport laws. By embedding coarse-grained cilia models within larger-scale solvers for two-layer coating, these hybrid approaches offer a principled route for coupling sub-micron simulations to organ-level clearance, enabling quantitative predictions of impaired mucociliary transport in diseases such as cystic fibrosis and primary ciliary dyskinesia.

At the microscopic end of the spectrum, modelling efforts focus on the alveolar and respiratory airways where surface tension, compliance and surfactant dominate. Here, the characteristic Reynolds number for air is below one and much smaller for the liquid coating the pulmonary tissues. Hence, inertia is negligible and the motion of air–liquid interfaces determines both ventilation and stability of respiratory airways and alveoli.

First-principle models of the multiphysics of acinar flow (see Fig. 2b) are reported in the literature using theoretical approaches [150], lattice-Boltzmann [151], or finite-volume methods [152] have revealed intricate patterns of recirculation and mixing during breathing cycles [104]. These flows enhance gas mixing beyond pure diffusion and may influence particle deposition at the alveolar scale. Coupling the local surface tension to surfactant concentration has shown how the lung maintains uniform inflation despite heterogeneity in alveolar size. Recent work integrates these alveolar models with capillary and lymphatic networks to study fluid clearance and oedema formation [153, 154]. Although direct validation is limited to *in vitro* microfluidic models and animal experiments, the qualitative agreement with physiological observations is encouraging.

Despite their realism, first-principle models face inherent trade-offs. High fidelity entails high cost and significant data requirements: accurate geometry, boundary conditions, and material parameters are indispensable. Their primary role is therefore not direct clinical deployment, but rather the generation of detailed mechanistic insight and reference solutions that can inform and calibrate reduced-order or data-driven models. In this sense, they remain the benchmark against which simplified approaches are evaluated. Their continuing development, incorporating adaptive meshing, GPU acceleration, and automated pipeline generation [155], moves towards narrowing the gap between research and clinical applicability.

### Reduced-order heuristic, network, and asymptotic models

Reduced-order models offer a complementary viewpoint to detailed CFD. They aim to capture the dominant physical mechanisms with substantially lower computational cost, but they span a wide range of spatial scales and modelling objectives. At the organ level, network and lumped-parameter formulations represent the airway tree through simplified resistance–compliance elements to reproduce global ventilation, pressure–flow relationships, and mechanical coupling. At smaller scales, asymptotic approaches derived from lubrication theory provide reduced descriptions of liquid-lined airway dynamics, including airway surface liquid redistribution, surfactant effects, and closure or reopening phenomena. Because these two classes address fundamentally different physical questions, they are discussed separately in the following subsections.

**Fig. 3:**
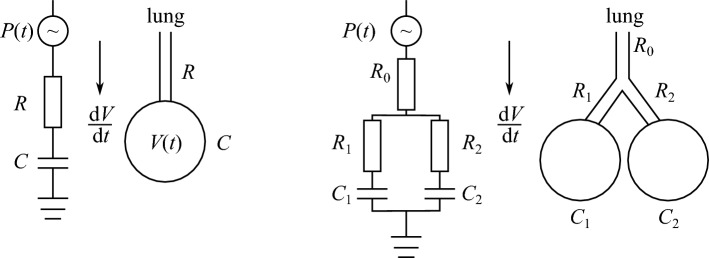
Two examples of lumped-parameter models, where *R* characterizes a resistance, *C* the compliance and *V*(*t*) denotes the lumped volume that changes in time. We refer to [129] for more details on the corresponding model equations, and a comparison with other lumped models

#### Network and lumped-parameter models

Instead of resolving the full three-dimensional velocity and pressure fields, some heuristic models replace the airway network by simplified elements governed by averaged relationships between pressure and flow. The earliest examples date back to the 1970s, when one-dimensional representations of the bronchial tree have been developed based on Poiseuille or Womersley theory. Each airway segment is treated as a compliant tube with a pressure drop $\Delta p = RQ + L\,\textrm{d}Q/\textrm{d}t$, where *R* and *L* represent hydraulic resistance and inertance, and the local wall mechanics define the compliance *C* [156]. Connecting thousands of such elements yields a lumped-parameter network that mimics the pressure–flow behaviour of the entire lung at minimal computational cost (see Fig. 3).

Because these 1D and 0D formulations neglect secondary and cross-sectional variations, their accuracy relies on suitable parameterization. Empirical correlations or data extracted from detailed CFD simulations are often used to assign effective resistances and compliances. The networks can then predict global quantities such as total airway resistance, regional ventilation, and flow partitioning among human lung lobes [93]. They also provide boundary conditions for three-dimensional models and can be extended to include multiphase flow and surfactant deposition [157], as well as liquid plug propagation and rupture [81, 158].

Reduced-order formulations have also been developed to incorporate microscale airway closure and surfactant effects into organ-scale simulations. For example, reduced-dimension models of recruitment and derecruitment dynamics capture the interaction between airway reopening mechanics and global ventilation distribution without resolving full interfacial flow fields [159]. Multiscale network approaches have further coupled surfactant transport and surface tension regulation with airway and acinar mechanics, enabling full-lung simulations that account for heterogeneous ventilation and mechanical ventilation scenarios [160, 161]. These approaches illustrate how detailed multiphase physics can be embedded into computationally tractable models suitable for large-scale or patient-specific studies.

The simplicity and reduced computational cost of 0D/1D models makes them particularly attractive for clinical applications. When integrated in network models, they can run in real time on small clusters and be calibrated directly from patient-specific data obtained by spirometry, plethysmography, or imaging. When combined with optimization algorithms, network models can estimate regional compliance or airway resistance from non-invasive measurements, aiding diagnosis of diseases such as asthma or COPD. Extensions including nonlinear wall mechanics, viscoelastic tissue coupling, and distributed alveolar units have further improved physiological realism. Nevertheless, their limitations are evident: secondary flows, turbulence, and interfacial phenomena are inherently excluded, and the topology of the network must be prescribed a priori.

While network representations efficiently capture global lung mechanics, they do not resolve microscale interfacial phenomena in liquid-lined airways, which are addressed by asymptotic thin-film models discussed next.

#### Asymptotic thin-film models

**Fig. 4:**
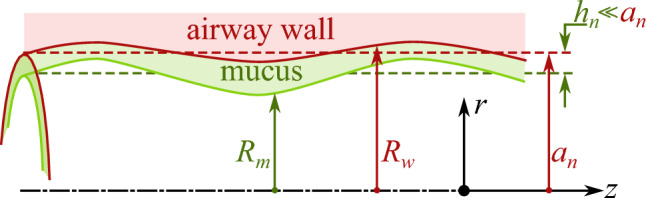
Schematic of an asymptotic model in which the mucus thickness $h_n$ is modelled as a thin film, i.e. $h_n\ll a_n$, and the mucus–air interface, as well as the airway wall are allowed to deform in the limit of small deformations, i.e. $\vert a_n-R_m\vert \ll a_n$ and $\vert a_n-R_w\vert \ll a_n$

Thin-film and lubrication theories have long provided the foundation for asymptotic reduced-order models of airway surface liquid, capturing liquid–plug formation, film rupture, and cyclic airway reopening under surface tension-dominated conditions [19, 37, 38]. Early analyses established how geometric perturbations, surfactant gradients, and wall compliance (see Fig. 4) modulate the Plateau–Rayleigh instability leading to closure [19, 36, 162], and how airway reopening generates large interfacial stresses relevant [74, 82, 163] to ventilator-induced injury. Subsequent extensions have incorporated oscillatory driving [25], surfactant transport [18, 21, 162, 164, 165], and nonlinear viscoelastic and viscoplastic effects [22, 24, 74, 75, 118, 144, 166], demonstrating that reopening fronts can impose substantial shear and normal stresses on epithelial cells. Complementary studies have examined surfactant-laden films, multilayer structures, and interactions between airway wall elasticity and fluid instability [64, 101, 164], clarifying how compliant or diseased airways alter the critical thickness thresholds for plug formation and rupture [38, 143, 163, 166].

More recent studies have incorporated rheological complexity [23, 167, 168] to better represent diseased mucus, introducing continuum models that account for shear-thinning, viscoelasticity, and yield-stress behaviour [169, 170]. These descriptions include fully viscoelastic mucus films driven by ciliary forcing, which demonstrate how polymeric stresses alter sliding, vortex formation, and net transport in ways that depart markedly from Newtonian lubrication predictions [24]. Complementing this, long-wave and thin-film models have been derived for yield-stress mucus layers [23, 171], showing that viscoplasticity increases the critical layer thickness for closure and delays plug formation, thereby quantifying the impact of mucus stiffening observed in chronic airway disease [23]. Together, these developments place classical thin-film theory within a broader multiphysics framework that integrates surfactant transport, wall deformability, viscoelasticity, and viscoplastic behaviour, providing an asymptotic foundation for understanding closure and reopening in both healthy and pathological lungs.

The applicability of lubrication-based thin-film models depends on geometric and dynamical scale separation. These asymptotic formulations are most accurate when the liquid-film thickness is small compared with the airway radius ($h_n/a_n \ll 1$), interfacial slopes remain moderate, and viscous and capillary forces dominate over inertia, typically corresponding to small capillary numbers and Reynolds numbers. Under these conditions, curvature variations occur primarily along the axial direction, and reduced one-dimensional descriptions capture the dominant physics of plug propagation, film deposition, and airway closure. However, when the liquid layer becomes comparable to the airway radius, when large interface deformations or breakup occur, or when inertial effects increase (e.g. during rapid reopening events), the assumptions underlying lubrication theory break down. In such regimes, fully resolved free-surface methods, such as volume-of-fluid or level-set approaches, are required to capture interface topology changes and three-dimensional flow structures accurately. These criteria provide practical guidance for selecting the appropriate modelling framework depending on airway geometry and flow conditions.

### Poroelastic and continuum tissue mechanics

**Fig. 5:**
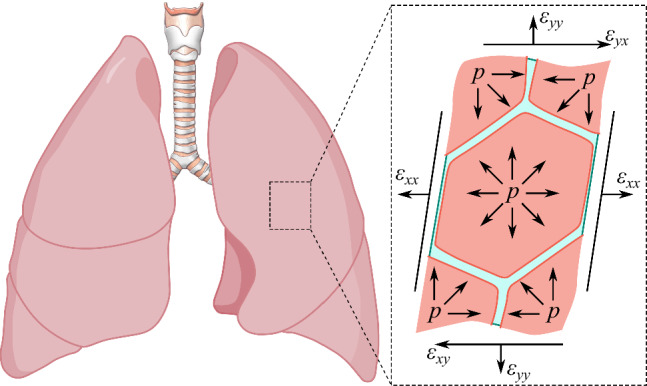
Schematic of a continuum poroelastic model (right) in which two deformable domains are identified to characterize the poroelastic microscopic properties of the lungs and finally describe their macroscopic dynamics (left)

The deformable nature of the parenchyma necessitates models that go beyond the purely fluid description of air motion. At the organ scale, the lung behaves as a porous, nearly incompressible continuum in which air and tissue coexist. Poroelastic models describe this behaviour through coupled equations for momentum and mass conservation in the solid and fluid phases [87]. Current numerical formulations allow for complex geometries, nonlinear material laws, and realistic pleural boundary conditions and can reproduce both the characteristic sigmoidal pressure–volume curve and the heterogeneous regional deformation observed in imaging during inflation and deflation [172, 173].

Continuum tissue models (see Fig. 5) have provided new insight into the distribution of mechanical stress and strain within the lung [174, 175]. Simulations show that regions near the diaphragm and costal surfaces experience larger deformations, consistent with imaging studies based on magnetic–resonance tagging and 4D-CT registration [172, 173]. When combined with gravity-dependent variations in stiffness or pleural pressure, poroelastic models explain ventilation gradients between dependent and non-dependent regions [58]. They also offer a framework for studying ventilator-induced lung injury, in which cyclic over-distension of alveoli leads to tissue damage [173, 175]. Incorporating damage or plasticity laws into the constitutive equations allows modelling of recruitment and hysteresis during repeated loading cycles [172, 173].

A challenge in continuum formulations is the identification of material parameters. Unlike engineering materials, biological tissues vary significantly between individuals and even between lung lobes. Inverse methods using imaging data or pressure–volume measurements have been developed to estimate local stiffness and permeability, but the results remain uncertain [87]. Despite these difficulties, continuum mechanics has become an indispensable link between micro-scale alveolar dynamics and macro-scale lung behaviour.

### Data-driven, PINN, and machine-learning approaches

The increasing availability of high-resolution imaging and large numerical databases has encouraged the use of machine learning to emulate or complement first-principle and mechanistic model simulations. Neural networks trained on first-principle datasets can predict flow fields, pressure distributions, or particle deposition patterns with orders-of-magnitude speed-ups compared with conventional solvers. These approaches rely on a range of machine-learning architectures and training paradigms.

A variety of machine-learning architectures have been explored in respiratory applications, depending on the nature of the data and the modelling objective. Convolutional neural networks are widely used for image-based tasks such as airway segmentation, geometric reconstruction, and regional ventilation estimation from CT or MRI data. Graph-based neural networks provide a natural framework for representing airway-tree topology and learning flow or resistance relationships across branching networks. Physics-informed neural networks incorporate governing equations directly into the training loss, enabling surrogate solutions of fluid or transport problems while enforcing physical constraints. Hybrid strategies combining data-driven components with reduced-order or mechanistic models are increasingly common, particularly when training data are limited or when interpretability is required. Training approaches typically range from supervised learning using high-fidelity simulation datasets to physics-constrained optimization and transfer learning across patient cohorts.

Reported performance gains vary depending on the application but often include orders-of-magnitude reductions in computational time compared with conventional numerical solvers once training is complete. For example, surrogate models trained on CFD or fluid–structure-interaction datasets can predict pressure–flow relationships or regional ventilation patterns within milliseconds, with errors typically in the range of a few per cent to tens of per cent depending on model complexity and training coverage. Data-driven approaches are particularly attractive for parameter estimation, uncertainty quantification, and real-time clinical decision support, where repeated model evaluations are required. However, predictive reliability remains strongly dependent on training-data quality, coverage of the parameter space, and consistency between training and deployment conditions, highlighting the importance of hybrid physics–data frameworks for robust applications.

Physics-informed neural networks (PINNs) enforce conservation laws within the training process, allowing interpolation between sparse measurements while maintaining physical plausibility [176, 177]. Gaussian-process regression and reduced-basis surrogates have been used to approximate the mapping between model parameters and global outputs such as airway resistance or tidal volume [100]. These tools facilitate uncertainty quantification and sensitivity analysis, which are essential for clinical reliability [178]. Beyond methodological diversity, their practical value depends on achievable accuracy and computational efficiency.

Despite their promise, physics-informed neural networks also present several challenges that currently limit their widespread application in respiratory modelling. Training can be sensitive to hyperparameter selection and loss-weight balancing, and convergence may become unstable when multiple coupled physical fields or stiff governing equations are involved. Enforcing boundary conditions on complex anatomical geometries remains difficult, particularly when high spatial accuracy is required near walls or interfaces. Moreover, although PINNs incorporate physical constraints, they often still require substantial training data or high-quality simulation results to achieve reliable predictive accuracy, especially in high-dimensional parameter spaces. These limitations imply that PINN approaches are presently most effective when used as surrogate models for well-characterized subproblems, or when combined with traditional numerical solvers in hybrid frameworks, rather than as fully standalone replacements for high-fidelity multiphysics simulations. Their use is therefore particularly attractive in reduced-order modelling, parameter estimation, and data-assimilation contexts where repeated evaluations are required.

Beyond surrogate modelling, recent developments in digital twin frameworks aim to create patient-specific, continuously updated computational replicas of the lung. Such approaches have demonstrated how high-fidelity biomechanical models, informed by imaging, physiology, and data assimilation techniques, can be calibrated to an individual’s anatomy and tissue properties to predict regional ventilation [179], mechanical strain [90], and disease progression. They integrate poroelastic or viscoelastic representations of parenchyma with parameter-estimation pipelines that reconcile simulations with clinical measurements such as pressure–volume curves or local deformation fields [90]. By embedding reduced-order or surrogate components within a full multiphysics model, these digital twins enable rapid re-evaluation as new data become available and offer a path towards personalized predictions of mechanical ventilation response, surgical outcomes, or therapeutic optimization [110].

**Fig. 6:**
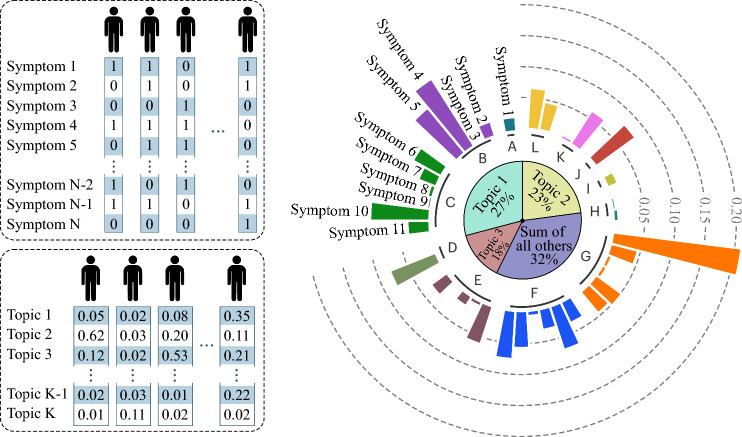
Example of data-driven approaches relying on purely statistical methods

In parallel with physics-based and hybrid approaches, purely statistical methods (see Fig. 6) built on large clinical databases have become an important complementary route for characterizing respiratory function and disease heterogeneity [180]. Leveraging electronic health records, imaging repositories, and longitudinal cohort studies, these models infer correlations between demographic factors, comorbidities, ventilation–perfusion metrics, and clinical outcomes without relying on explicit mechanistic equations [181]. Techniques such as latent-variable modelling [182], cluster analysis [183], and probabilistic topic modelling [184] have revealed distinct respiratory subphenotypes in conditions ranging from COPD to post-viral syndromes, while regression and survival models link routinely collected measurements (spirometry, CT attenuation, blood gas values) to increase of risk or treatment response [185]. Because these frameworks can incorporate tens of thousands of patients, they capture population-level variability that is difficult to reproduce in mechanistic models alone and provide statistical priors for parameter calibration, model selection, or patient stratification [180, 186]. Moreover, machine learning is routinely used to extract information directly from medical images. Convolutional neural networks can segment airway trees, estimate local wall thickness, and infer functional parameters such as ventilation or perfusion from dynamic CT or MRI [187]. Combining these image-based estimates with mechanistic models yields hybrid approaches that balance physical interpretability and data-driven flexibility. While still developing, such tools are expected to play a growing role in personalizing lung models and their clinical application. Table 2 summarizes data-trained approaches and their application.

**Table 2:** Representative data-trained approaches in respiratory modelling

Application	Architecture	Target Quantity	Training Data	Typical Benefit
Airway segmentation and network reconstruction	Convolutional neural networks (CNN)	Airway geometry CT/MRI	Medical imaging datasets	Automated geometry extraction
Flow and pressure surrogates	Fully-connected neural network / Reduced-order machine learning	Pressure–flow relationships, impedance	CFD or FSI simulations	Millisecond prediction time
Ventilation distribution prediction	Graph neural networks (GNN)	Regional airflow or resistance in airway trees	Network simulations or imaging- derived data	Organ-scale inference with reduced cost
Physics- informed flow modelling	Physics- informed neural networks (PINN)	Velocity, pressure, transport fields	Governing equations + sparse data	Reduced need for labelled data
Parameter estimation and inverse problems	Hybrid ML + optimization	Tissue properties, boundary conditions	Experimental or clinical measurements	Accelerated parameter identification
Clinical decision support / digital twins	Surrogate or ensemble models	Patient- specific response prediction	Multiscale simulations + clinical data	Near– real-time evaluation

### Graphical summary of mechanistic approaches

**Fig. 7:**
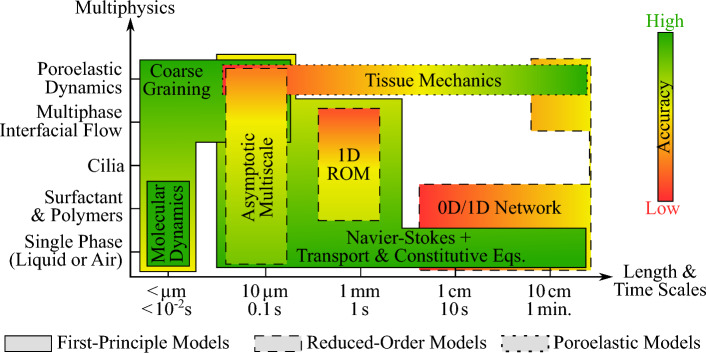
Summary of mechanistic modelling approaches for pulmonary fluid mechanics: first-principle models (solid lines), reduced-order models (dashed lines), and poroelastic models (dotted lines). An estimate of the accuracy is depicted by colours from red (low accuracy) to green (high accuracy). The term “Multiphysics” is not intended to represent a quantitative count of physical effects, but rather the increasing level of physical complexity and details incorporated in the models

Figure 7 summarizes the principal classes of deterministic models reviewed above, their characteristic spatial and temporal scales, and their expected ranges of predictive accuracy. These classes span from highly resolved first-principle simulations to reduced-order 1D network models and lumped-parameter representations, each trading physical detail for computational tractability. Statistical, data-driven, and machine-learning approaches are not shown explicitly, as they can be layered onto any of these deterministic frameworks (either by providing surrogate closures, accelerating parameter estimation, or enabling real-time prediction), provided that sufficiently rich training datasets are available. Their flexibility allows them to complement rather than replace mechanistic models, enhancing interpretability or computational efficiency where direct simulation would otherwise be prohibitive.

## Coupling strategies and multiscale frameworks

The respiratory system operates through tightly coupled physical and physiological mechanisms: airflow in the conducting airways drives tissue deformation; parenchymal strain redistributes ventilation; capillary and lymphatic flows regulate gas and fluid exchange; and all of these interact through the thoracic boundary conditions. Moreover, surfactant and non-Newtonian mucus layers modulate interfacial and wall stresses and stabilize or destabilize small airways. These multiphase effects feed back onto flow resistance, airway mechanics, and biochemical transport. Capturing these interdependencies requires coupling distinct physical domains, as well as spatial and temporal scales. Two complementary approaches are currently in use: *hierarchical coupling*, in which detailed models of selected regions interface with simplified representations elsewhere, and *concurrent coupling*, in which multiple physical fields are solved simultaneously in overlapping regions. This section summarizes both approaches and the numerical strategies that make them tractable.

Concrete implementations of multiscale coupling span several levels of description. A widely used hierarchical strategy couples three-dimensional CFD in the upper airways to one-dimensional bronchial-tree networks, thereby providing physiologically consistent distal boundary conditions while retaining computational tractability. In such 3D–1D formulations, pressures and flow rates are exchanged across the interface (often through impedance-based or iterative matching) to enforce mass conservation and consistency of the pressure–flow relationship. At the organ level, additional coupling to lumped (0D) elements is frequently introduced to represent global compliance, chest-wall mechanics, or patient-specific pressure–volume behaviour. Beyond purely aerodynamic coupling, hybrid frameworks also link airway FSI with continuum or poroelastic descriptions of the parenchyma, and embed reduced thin-film or recruitment/derecruitment submodels into network solvers to account for small-airway closure and reopening. Such 3D–1D–0D implementations have become a standard paradigm for integrating detailed airway aerodynamics with organ-scale lung mechanics. Collectively, these examples illustrate how coupling provides a practical bridge between local, high-fidelity physics and organ-scale predictions that are otherwise inaccessible within a single modelling level. These coupling strategies provide the mechanisms through which the modelling hierarchy introduced in Sect. 3 can be combined into unified predictive frameworks.

### Hierarchical and multiscale coupling

Hierarchical coupling exploits the intrinsic separation of spatial and physical scales present in the respiratory system by combining models of different fidelity within a single computational framework. High-resolution three-dimensional simulations are typically restricted to the trachea and central bronchi, where complex flow features such as separation, secondary motions, and turbulence are most pronounced, while distal generations are represented using reduced-order or lumped formulations. In a common implementation, each outlet of the 3D CFD domain is connected to a one-dimensional airway branch governed by an unsteady Poiseuille or Womersley relationship, and terminal acinar regions are replaced by zero-dimensional compliance elements [93]. Information exchange occurs through pressure and flow at the interfaces: the CFD solver provides instantaneous outlet flow rates, which determine boundary conditions for the 1D network, while the network returns updated pressures to enforce mass conservation and impedance matching across scales.

This 3D–1D–0D hierarchy reproduces the physiological impedance of the downstream airways while maintaining computational tractability. Recent implementations include automated generation of distal airway networks using morphometric scaling laws and statistical reconstruction techniques, as well as adaptive refinement in regions of interest [58]. When coupled with vascular or perfusion models, the same framework naturally extends to ventilation–perfusion simulations, linking airflow in the bronchial tree to blood flow in the pulmonary circulation. Such multiscale models enable predictions of regional ventilation, perfusion, and gas exchange and have shown encouraging agreement with imaging measurements in both healthy and diseased lungs [54].

Hierarchical coupling also applies beyond geometrical decomposition, encompassing temporal and physical-scale separation [188]. Processes evolving on disparate time scales—such as rapid airflow fluctuations, surfactant transport, and slower tissue deformation or perfusion—may be advanced using different temporal resolutions within operator-splitting or staggered integration frameworks. This approach preserves numerical stability while reducing computational cost; for example, a single breathing cycle may require hundreds of time steps for airflow but only a few updates for tissue mechanics. Similar multiscale concepts appear in models of mucociliary clearance, where ciliary forcing is represented through effective boundary conditions encoding the metachronal wave, while the mucus layer is treated as a viscoelastic film whose evolution occurs on longer time scales. Recent work demonstrates that such hierarchical representations can capture the interaction between ciliary beat coordination and mucus transport without explicitly resolving individual cilia [24]. Overall, hierarchical coupling provides a pragmatic strategy to integrate local high-fidelity physics with organ-scale behaviour while maintaining computational feasibility.

### Concurrent multiphysics coupling

In contrast to hierarchical approaches, concurrent coupling resolves multiple physical processes simultaneously within the same spatial region, typically without separating domains by fidelity or scale. In concurrent coupling, the different physical fields (fluid, structure, surfactant, or non-Newtonian stresses) are solved within the same spatial domain, often sharing common meshes or interfaces. Fluid–structure interaction and poroelastic formulations are typical examples. The coupling conditions enforce continuity of velocity and stresses at the air–tissue interface, ensuring conservation of mass and momentum. Two main numerical paradigms exist. *Monolithic* approaches assemble the fluid and solid equations into a single global system, guaranteeing strong coupling but at high memory cost. *Partitioned* approaches, by contrast, iterate between fluid and solid solvers, exchanging boundary data until convergence. Explicit coupling is computationally cheaper but may suffer from numerical instability due to the significant difference between tissue and air density [87], often referred to as the added-mass effect.

Coupling may also involve distinct continua, as in poroelastic models where the tissue skeleton and interstitial air coexist within a single domain. In this case, the coupling terms represent drag and volume exchange rather than interface continuity. Such models naturally handle topological changes and allow simulation of alveolar recruitment or collapse without explicit tracking of moving boundaries [58]. Hybrid poroelastic–FSI formulations, combining Eulerian and Lagrangian descriptions, have recently been proposed to capture both large deformations of the parenchyma and unsteady airway flow [86] , thereby enabling consistent treatment of airway mechanics and tissue deformation within a unified framework.

Concurrent coupling also extends to multiphase transport and rheological phenomena occurring within the airway lumen. A related challenge arises in reconciling microscale models of mucus with continuum descriptions used in airway simulations. Recent works have shown that the nonlinear behaviour of airway mucus arising from polymer entanglement, crosslinking, and hydration gradients can be captured by microscale constitutive models that reproduce shear-thinning, yield-stress behaviour, and elastic recoil [102, 189]. These high-fidelity formulations have been systematically coarse-grained to produce tractable continuum laws for plug movement, rupture, and airway closure [102]. These efforts demonstrate how integrating microscale rheology with continuum constitutive laws enables more reliable predictions of mucus transport, plug fragmentation, and clearance under physiological and pathological conditions, implying, however, a significantly higher computational cost.

Overall, concurrent coupling provides the most physically comprehensive representation of interacting processes, but at the expense of increased numerical complexity and computational cost compared with hierarchical strategies.

### Emerging hybrid frameworks

Beyond purely physics-based coupling strategies, emerging respiratory models increasingly adopt hybrid formulations that combine mechanistic solvers with data-driven or reduced-order components. Machine-learning surrogates can replace expensive submodels within a coupled simulation, for example by approximating distal airway impedance, tissue constitutive responses, or subgrid multiphase processes based on prior high-fidelity computations. Such surrogates maintain physical coupling through interface conditions but dramatically reduce computational cost. Similarly, reduced-basis methods and proper-orthogonal-decomposition expansions approximate the solution manifold of detailed models, enabling near real-time evaluation during clinical decision-making [190, 191] while preserving the dominant dynamical behaviour of the underlying multiphysics system.

These hybrid approaches naturally complement the hierarchical and concurrent coupling strategies described above. Hybridization also extends beyond numerical acceleration to the coupling of multiple physiological subsystems. Another frontier lies in coupling across biological systems. The alveolar–capillary interface connects the respiratory and cardiovascular domains; interstitial and lymphatic flows link to systemic fluid balance [153, 154]. Coupled respiratory–circulatory models are beginning to quantify how cardiac output, blood oxygenation, and pulmonary vascular resistance interact with ventilation patterns [28]. Integration with thermoregulation and metabolic models could eventually produce a comprehensive simulation of oxygen transport from atmosphere to mitochondria [186, 192].

These developments suggest a convergence between multiscale physiological modelling and hybrid computational paradigms, in which physics-based coupling provides mechanistic consistency while data-driven components enhance computational tractability and enable patient-specific personalization.

## Summary and perspectives

The study of pulmonary fluid mechanics has evolved from idealized analytical descriptions of airway resistance to a comprehensive, multiscale view of the respiratory system as a living, multiphysics system. The preceding sections have traced this development from first-principle formulations, through reduced-order and continuum models, to hybrid and data-driven frameworks. Each level of representation considers a different facet of lung function: detailed simulations reveal flow structures and stress distributions; network and poroelastic models capture global ventilation and tissue deformation; and statistical or machine-learning surrogates bridge the gap to real-time clinical applications. What unites these diverse approaches is a recognition that the lung cannot be decomposed into independent parts as its behaviour arises from the dynamic coupling of air, liquids, and tissues across scales.

Developing such a feedback loop also demands new infrastructures for data curation, standardization, and sharing. Multicentre clinical cohorts provide increasingly rich datasets, but aligning them with simulation frameworks requires harmonized anatomical segmentations, comparable ventilatory protocols, and consistent metadata describing patient status. On the modelling side, interoperable software pipelines and reproducible workflows are essential for ensuring that predictions can be verified and audited across institutions. As regulatory bodies begin to consider in silico evidence in device design or therapy planning, the ability to demonstrate traceability, from raw clinical recordings to processed model inputs and validated outputs, will be crucial. Without these integrative efforts, computational models will remain scientifically informative but fall short of routine clinical impact.

From a scientific standpoint, the most significant challenge remains validation and accurate multiphysics multiscale prediction. While computational fidelity continues to increase, experimental data at matching resolution are scarce. Bridging this gap will require coordinated development of imaging modalities and measurement protocols that can supply quantitative boundary conditions and benchmark data for multiphysics models. Recent advances in four-dimensional CT, phase-contrast MRI, and synchrotron imaging offer promising routes, but systematic cross-comparison between experiments and simulations is still rare. Community-wide benchmark initiatives, analogous to those long established in aerodynamics or haemodynamics, would accelerate convergence towards reproducible standards.

Another priority is the efficient integration of models into clinical workflows. In their current form, most high-fidelity simulations remain research tools that demand substantial computing resources and expertise. To become clinically useful, they must deliver actionable results, such as ventilation heterogeneity maps, airway resistance distributions, or aerosol deposition fractions. Moreover, all this shall be done within times compatible with medical decision-making. Reduced-order and machine-learning surrogates will be indispensable in this regard, enabling near-instantaneous predictions once trained on a library of detailed solutions. However, validation must ensure that these surrogates preserve the physics and remain interpretable to clinicians.

Clinical examples already demonstrate the potential of coupled models. In chronic obstructive pulmonary disease, simulations have clarified how airway narrowing and loss of parenchymal recoil lead to regional gas trapping and ventilation–perfusion mismatch. In cystic fibrosis, models incorporating non-Newtonian mucus rheology reproduce impaired mucociliary clearance and identify threshold viscosities beyond which transport fails [24, 149]. During mechanical ventilation of ARDS patients, FSI and poroelastic simulations predict regions of high stress concentration that correlate with ventilator-induced lung injury, suggesting strategies for protective ventilation [58, 86]. For neonatal respiratory distress, thin-film and surfactant models help optimize surfactant replacement therapy [19, 30]. Aerosol-transport simulations are guiding the design of inhalers and the selection of particle sizes and breathing patterns to maximize therapeutic deposition [16, 73]. These examples illustrate how modelling has progressed from theory to practical clinical relevance.

Conceptually, coupling strategies embody the trade-off between fidelity and feasibility. Hierarchical approaches leverage physical insight to distribute computational effort where it matters most; concurrent approaches ensure strict consistency of coupled fields. Future progress will rely on flexible frameworks that can switch between these modes as dictated by accuracy requirements and available data. Ultimately, predictive lung modelling will depend not only on solving the right equations but on coupling them in ways that respect both the physics of the organ and the practical constraints of computation and clinical use. Several research directions are identified:

- *Quantitative Validation and Benchmarking.* Establish standardized geometries, boundary conditions, and data repositories that allow cross-comparison of models and algorithms. Reference cases covering laminar, transitional, and creeping flow regimes would provide a foundation for reproducible progress.
- *Multiphysics Integration.* Extend existing airflow and acinar flow models to include perfusion, gas exchange, moisture transport, realistic non-Newtonian models, and surfactant dynamics, together with cilia beating and cellular response. Coupled respiratory–circulatory simulations could quantify how alterations in blood flow or cardiac output affect ventilation and oxygenation.
- *Parameter Identification and Uncertainty Quantification.* Apply Bayesian and multi-fidelity methods to estimate patient-specific parameters from imaging and functional data, propagating uncertainty to predicted outcomes.
- *Real-Time and Personalized Modelling.* Combine reduced-basis and machine-learning techniques to produce fast, adaptive “digital twins” of patient-specific lungs capable of updating in real time as new data become available. Such twins could predict the response to interventions, aid in ventilator setting optimization, and provide early warnings of deterioration.
- *Interdisciplinary Collaboration.* Encourage closer interaction between engineers, physiologists, and clinicians. Mechanistic insight from models can inform clinical interpretation, while clinical data can refine and validate the models.

Moving towards predictive and clinically actionable respiratory models requires addressing several unresolved quantitative challenges:

- Can multiscale models predict regional ventilation and perfusion in diseased lungs within clinically relevant accuracy ($\approx 10\%$) across patients?
- What minimum imaging and physiological data are required to construct patient-specific digital twins with clinically useful predictive capability?
- Can multiphysics models quantitatively predict mucus plug formation and rupture pressures during mechanical ventilation, and identify safe operating regimes?
- To what extent can reduced-order or hybrid physics–data approaches maintain predictive accuracy while enabling near-real-time clinical simulation?

In conclusion, lung fluid mechanics has matured into a multidisciplinary field where classical fluid dynamics, continuum mechanics, and data science converge. The task now is not simply to simulate more accurately but to simulate more meaningfully: to connect physical quantities such as pressure, stress, and flow with clinical indicators such as compliance, oxygenation, and clinical risks. Achieving this will transform models from descriptive tools into predictive instruments of patient care.
